# Antimicrobial metallic copper surfaces kill *Staphylococcus haemolyticus* via membrane damage

**DOI:** 10.1002/mbo3.2

**Published:** 2012-03

**Authors:** Christophe Espírito Santo, Davide Quaranta, Gregor Grass

**Affiliations:** 1 Department of Life Sciences, Faculty of Sciences and Technology, University of Coimbra and Marine and Environmental Research Center (IMAR-CMA)3001-401 Coimbra, Portugal; 2 School of Biological Sciences, University of Nebraska-LincolnNE 68588

**Keywords:** Genotoxicity, membrane damage, metallic copper toxicity, *Staphylococcus*

## Abstract

Recently, copper (Cu) in its metallic form has regained interest for its antimicrobial properties. Use of metallic Cu surfaces in worldwide hospital trials resulted in remarkable reductions in surface contaminations. Yet, our understanding of why microbes are killed upon contact to the metal is still limited and different modes of action have been proposed. This knowledge, however, is crucial for sustained use of such surfaces in hospitals and other hygiene-sensitive areas. Here, we report on the molecular mechanisms by which the Gram-positive *Staphylococcus haemolyticus* is inactivated by metallic Cu. *Staphylococcus haemolyticus* was killed within minutes on Cu but not on stainless steel demonstrating the antimicrobial efficacy of metallic Cu. Inductively coupled plasma mass spectroscopy (ICP-MS) analysis and in vivo staining with Coppersensor-1 indicated that cells accumulated large amounts of Cu ions from metallic Cu surfaces contributing to lethal damage. Mutation rates of Cu- or steel-exposed cells were similarly low. Instead, live/dead staining indicated cell membrane damage in Cu- but not steel-exposed cells. These findings support a model of the cellular targets of metallic Cu toxicity in bacteria, which suggests that metallic Cu is not genotoxic and does not kill via DNA damage. In contrast, membranes constitute the likely Achilles’ heel of Cu surface-exposed cells.

## Introduction

Metallic copper (Cu) surfaces have excellent antimicrobial properties against a variety of different microorganisms from different domains of life (Grass et al. 2011). As such, Cu touch surfaces can be expected to support existing hygiene-increasing procedures in public places including hospitals. Indeed, in worldwide hospital trials non-Cu surfaces in frequent contact with patients and staff were replaced with their Cu counterparts. This novel use of metallic Cu resulted in diminishing bacterial surface-loads up to 90% as compared to controls (Casey et al. 2010; Mikolay et al. 2010). Recently, molecular mechanisms that result in rapid killing of Cu surface-exposed bacteria and yeasts were studied. Both groups of organisms are killed by a sharp shock of extreme and immediate Cu ion overload combined with extensive membrane and envelope damage. Importantly, exposure to metallic Cu did not result in genotoxicity. Actually, similar low mutation rates were observed in cells from Cu and control surfaces (Espirito Santo et al. 2008; Quaranta et al. 2011).

While it was previously reported that Staphylococci were inactivated by both moist and dry Cu surfaces (Mehtar et al. 2007; Michels et al. 2009; Espirito Santo et al. 2010), the molecular mode-of-action leading to complete kill remained controversial. An alternative model that differs from the mode-of-action model involving membrane damage as outline above and in Airey and Verran (2007) predicts that the thick Gram-positive cell walls of Staphylococci were significantly different from that of *Escherichia coli*, other Gram-negative bacteria and yeasts requiring a different mechanism of kill. Indeed, Keevil and coworkers reported that DNA inside Cu-exposed *Staphylococcus aureus* cells was degraded causing cell death. Yet, the authors observed only little negative effect on cytoplasmic membrane integrity (Weaver et al. 2010).

Here, we demonstrated that killing on metallic Cu of *S. haemolyticus*, as a model organism from the staphylococcal group of notorious pathogens, follows the same rules of inactivation by antimicrobial Cu surfaces as observed for other microbial species.

## Material and Methods

### Bacterial strains and growth media

The strain used in this study was *S. haemolyticus* NRRL B-14755 (Schleifer and Kloos 1975). It was grown in R2A broth (Difco BD, Franklin Lakes, NJ USA), at 30°C with rotary shaking (250 rotation per minute [RPM]) until stationary growth phase (approximately 16 h of incubation). Bacto Agar (Difco BD, Franklin Lakes, NJ USA) was added at 15 g × L^−1^ for solid media.

### Contact killing assay on metal surfaces

Metal surfaces used in this study were 2.5 × 2.5 cm Cu coupons (C11000, 99.9% Cu) or stainless steel control coupons (AISI 304, approximately 67–72% Fe, 17–19.5% Cr, 8–10.5% Ni). Coupons were provided by the International Copper association (New York City, NY USA). All Cu-alloy coupons were treated prior to each experiment to standardize the surface properties. Coupons were incubated for 30 sec in 3% (w/v) NaOH solution at 70°C and rinsed in distilled water. After transfer into 10% (v/v) sulfuric acid solution for 5 sec at room temperature (23°C) coupons were immediately washed with distilled water. All coupons, Cu and stainless steel, were disinfected and cleaned by immersion in ethanol and kept in a sterile container. To prevent surface reoxidation cleaned coupons were not flamed after immersion in 95% ethanol.

For determination of the survival of cells on dry metal surfaces, cultures were concentrated 10-fold and tested as described in Espirito Santo et al. (2011) with minor changes. Aliquots of 10^6^ cells were streaked out on coupons using sterile cotton swabs. All samples dried completely within 5 sec after contact with the surfaces. Unless indicated otherwise, this time point is considered “0” or t_0_ throughout this study. Cell-laden coupons were incubated in sterile Petri dishes at 23°C for different times to avoid contamination from the laboratory environment. Coupons were transferred into 10-mL ice-cold phosphate-buffered saline (PBS) with approximately 20 glass beads (2 mm, Sigma-Aldrich, St. Louis, MO USA) (PBSG buffer). Samples were vortexed for 1 min, diluted in PBS buffer and plated on LB agar. Surviving bacteria were counted as colony forming units (CFU) using an automatic counter (Acolyte, Synbiosis, Cambridge UK) and the associated software (Version 2.0.8).

### Mutagenicity assay

The occurrence of mutations as the emergence of D-cycloserine resistant clones in Cu surface-exposed cells and controls was tested as described previously (Espirito Santo et al. 2011). In short, cells were applied for 5 sec to the surface of the metal coupons (a time period of exposure shorter than required for killing), removed with PBS as described above and concentrated. Cells were spread on solidified minimal medium with glycerol as sole carbon source for determination of total CFU and on minimal media containing glycerol and 80 μg × ml^−1^ D-cycloserine (Sigma-Aldrich, St. Louis, MO USA) to select for D-cycloserine resistant mutants. Colonies assumed to have originated from mutations in the *aapA* gene inactivating D-cycloserine uptake, were counted after 24 h of incubation. The percentage of *aapA* mutants was calculated by dividing the number of CFU of *aapA* mutants by the total number of CFU. For comparison, cells were exposed for the same period of time on stainless steel or on stainless steel with 0.9% (w/v) formaldehyde as a known mutagen. To assess if groups of data were statistically different from each other, *t*-test was performed with data of Cu-, stainless steel-, or formaldehyde-exposed cells on stainless steel (positive control). The two-tailed probability values (*P*) were ≤ 0.05.

### Inductively coupled plasma mass spectroscopy (ICP-MS) analysis

The uptake of solubilized Cu ions from metallic surfaces was determined as described by Espirito Santo et al. 2011). For this, cells were spread directly on surfaces of Cu coupons as described above. At various time points cells were removed from surfaces and excess Cu was removed by washing with ice-cold PBS-buffer containing 20 μM EDTA for chelating externally bound Cu. Acid-mineralized samples were diluted to adjust to a final concentration to 5% v/v of nitric acid. As internal standard Gallium (Ga(NO_3_)_3_) was added at a final concentration of 50 ppb. Element analysis was performed using an Agilent ICP-MS model 7500cx (Agilent, Santa Clara, CA USA) operating with a collision cell with a flow of 3.5 mL × min^−1^ of H_2_ and 1.5 mL × min^−1^ of He. Data for each sample were accumulated in triplicate for 100 msec. For quantification an external calibration curve was recorded with Gallium in 5% nitric acid. Initial cell numbers were determined by plating as described above.

### Live/dead staining to evaluate membrane damage

A live/dead staining technique was employed to differentiate cells on Cu and control surfaces with undamaged and damaged, permeable membranes (LIVE/DEAD®*Bac*Light™ Bacterial Viability Kit, Invitrogen, Grand Island, NY USA) as described earlier (Espirito Santo et al. 2011). Stained cell samples were examined by fluorescence microscopy (λ_Ex_ = 488/543 nm, λ_Em_ = 522/590 nm) under oil immersion using an inverted confocal microscope (Olympus, IX 81, Olympus America, Center Valley, PA USA). For the dye SYTO® 9, the laser used was Argon 488 nm and for propidium iodide HeNe_G 543 nm. Image capture software was Fluoview 500 (Olympus America, Center Valley, PA USA).

### Visualization of labile intracellular Cu(I) pools

Coppersensor-1 (CS1, 8-[*N*,*N*-Bis(3′,6′-dithiaoctyl)-aminomethyl]-2,6-diethyl-4,4-difluoro-1,3,5,7-tetramethyl-4-bora-3*a*,4-*a*-diaza-*s*-indacene) is a membrane permeable fluorescent dye, which after selectively binding to Cu(I) increases its red fluorescence by 10-fold. CS-1 was synthesized (Miller et al. 2006) and employed to quantify changing intracellular Cu(I) concentrations as described in Espirito Santo et al. (2011). Cu accumulation within cells was examined under oil-immersion (λ_Ex_ = 543 nm, λ_Em_ = 555–600 nm) with an upright fluorescence microscope (Olympus AX70, Olympus America, Center Valley, PA USA). Image capture software was Fluoview 500 (Olympus America, Center Valley, PA USA) and the laser used was HeNe_G 543.

## Results

### *Staphylococcus haemolyticus* is quickly killed on dry metallic Cu

**Figure 1:**
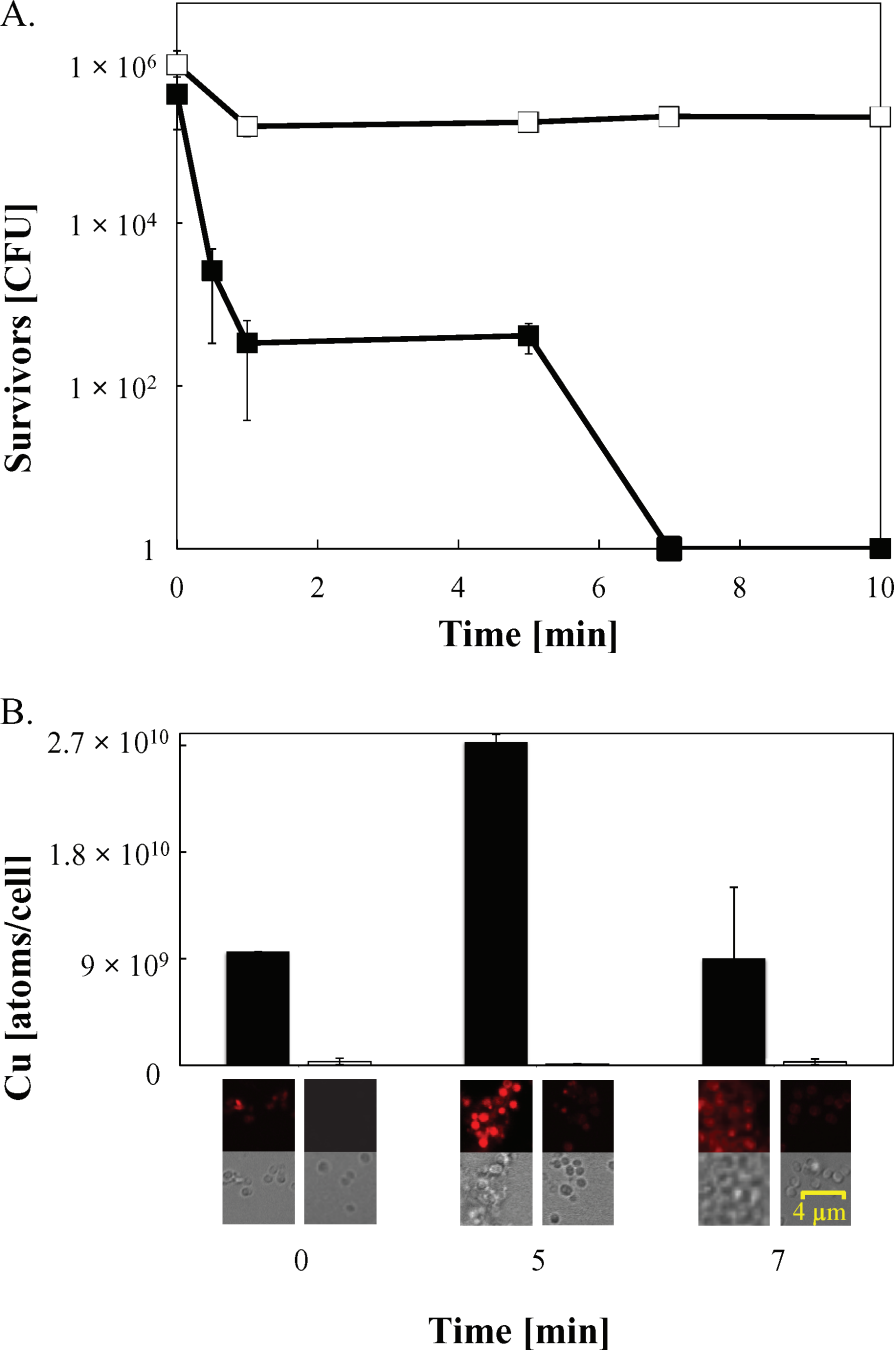
*Staphylococcus haemolyticus* is rapidly killed on dry metallic copper (Cu) surfaces and cells accumulate large amounts of Cu. Cells of *S. haemolyticus* were exposed to dry metallic Cu surfaces (▪) or stainless steel (□) for the indicated times, removed, washed, and plated on solidified growth media. Survivors were counted as colony forming units (CFU) (A). Parallel samples (black bars, from Cu; white bars, from stainless steel) were mineralized and subjected to ICP-MS analysis for determination of cellular Cu content (B, upper panel) or were stained with the Cu(I)-specific fluorescent dye coppersensor-1 and subjected to fluorescence microscopy (B, lower panel). Shown are averages of triplicate experiments with standard deviations (error bars) and representative phase contrast and fluorescence microscopy images, respectively.

Previous studies tested Staphylococci on moist Cu (Airey and Verran 2007; Weaver et al. 2010) or investigated long-term survival on dry Cu surfaces (Espirito Santo et al. 2010). Here we tested in a time course exposure experiment the killing kinetics of *S. haemolyticus* on dry Cu. Cells were grown, exposed to Cu or stainless steel control surfaces, removed, and survivors counted. Cells were largely unaffected by contact to stainless steel for the duration of the experiment. However, on Cu all 10^6^ cells were killed after 7 min (Fig. 1A) demonstrating that *S. haemolyticus* can be inactivated within minutes on dry Cu.

### Cells rapidly accumulate large amounts of dissolved Cu from surfaces

We employed the qualitative Cu-specific fluorescent dye Coppersensor-1 and quantitative ICP-MS to follow the degree and kinetics of Cu ion uptake from the surfaces into cells. Cells even immediately removed from Cu (t_0_) had accumulated about 10 billion Cu atoms (Fig. 1B, upper panel). After 5 min, maximum concentrations of Cu were reached and at 7 min, the time when all cells were killed, the concentrations declined again. In contrast Cu concentrations in cells from stainless steel remained constant at low levels throughout (at about 2 × 10^8^). Concentrations of other metals were also measured by ICP-MS (data not shown). For instance, concentrations of zinc or iron remained very similar in cells exposed to stainless steel or Cu, respectively.

Cells stained with Coppersensor-1 fluoresced brightly red when exposed to Cu surfaces for 5 min, at time by which about 99.9% of the cells have succumbed to Cu toxicity (Fig. 1B, lower panel). In contrast, cells immediately removed from Cu (t_0_) or from stainless steel fluoresced weakly indicative of low Cu (Fig. 1B, lower panel). The apparent conflicting data (Coppersensor-1/ICP-MS) for Cu exposed cells at t_0_ can easily be explained by the thick peptidoglycan of the cells. This polymer likely accumulated and slowed down the Cu ions diffusing toward the cytoplasm, where Coppersensor-1 was located.

### Exposure to metallic Cu is not genotoxic to *Staphylococcus*

**Figure 2:**
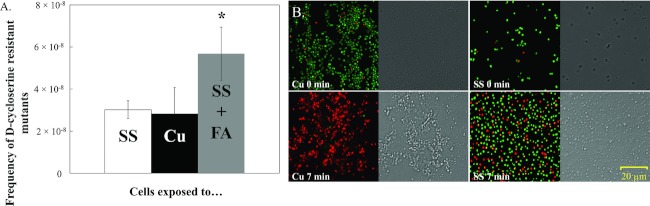
Exposure to metallic copper (Cu) surfaces does not promote mutations but causes membrane damage. Cells of *Staphylococcus haemolyticus* (10^10^ cells per sample) were exposed for 5 sec to Cu, stainless steel, or 0.25% (wt/vol) of the mutagen formaldehyde (CH_2_O) + stainless steel surfaces. Cells were washed from surfaces, concentrated, and spread on solid media containing 80 μg × ml^−1^D-cycloserine. D-cycloserine is bacteriostatic and colonies arise from inactivating mutations in the gene of the D-cycloserine uptake-permease AapA (A). Cells were exposed to metal surfaces for 0 or 7 min, removed, washed, subjected to Live/dead staining, and observed by fluorescence microscopy (B). Live bacteria with undamaged membranes fluoresce green, cells with damaged membranes fluoresce red. Shown are averages of triplicate experiments with standard deviations (error bars, A) or representative micrographs from three independent experiments with similar results (B). The *asterisk* denotes significantly (*P* ≤ 0.05, *t* -test) different values in the mutagen formaldehyde-treated controls.

Because genotoxicity caused by metallic Cu is controversial in Staphylococci, we next investigated if exposure to metallic Cu caused an increase in mutations in *S. haemolyticus*. For this, cells were exposed to Cu or stainless steel for 5 sec (before onset of massive cell death), washed, and plated onto solid media containing 80 μg/mL D-cycloserine. D-cycloserine interferes with cell wall biosynthesis and cells can only grow in its presence when a mutation event in the *aapA* gene has occurred, inactivating the D-serine/D-alanine/glycine transporter AapA by which D-cycloserine is likely taken up. Exposure to both Cu and stainless steel resulted in very similar numbers of resistant mutants, clearly indicating that metallic Cu did not increase mutation events in exposed cells (Fig. 2A). In contrast, when the known mutagen formaldehyde was added to cells before exposure to stainless steel, significant higher mutant numbers (*t*-test, *P* ≤ 0.05) were observed.

### Contact to metallic Cu damages *Staphylococcus* membranes

Dry Cu surfaces did not cause mutation damage to the DNA (Fig. 2A). An alternative explanation for cell death after contact to metallic Cu might be lethal membrane damage. We investigated membrane damage using viability staining (LIVE/DEAD® *BacLight*™ Bacterial Viability Kit, Invitrogen, Grand Island, NY USA). One dye (Syto 9^©^) stains DNA in all cells, those with intact and those with compromised membranes, green. The other dye (propidium iodide) can only enter cells with damaged membranes and stains DNA red. Cells in contact with Cu at *t*_o_ had largely undamaged membranes and stained green (Fig. 2B) but virtually all cells had membrane damage (red) after 7 min. Conversely, the majority of cells on stainless steel remained green, that is, had undamaged membranes throughout the experiment. The increase in numbers of damaged (red) cells correlated well with the killing kinetics (Fig. 1A) in which also some death on stainless steel was observed. This background damage and lethality is likely owed to desiccation events occurring on these dry surfaces. However, the stainless steel controls clearly indicate that the killing on Cu is not due to simple desiccation but rather mediated by contact with the Cu surfaces.

## Discussion

Overall, our results suggested that death in *S. haemolyticus* after contact to antimicrobial metallic Cu coincided with membrane damage and that lethality was not caused by genotoxicity. As such, the Gram-positive Staphylococci were not very different in the events leading to killing from the Gram-negative *E. coli*, *Deinococcus radiodurans* from the bacterial *Deinococcus–Thermus* phylum (Espirito Santo et al. 2011) or the yeast *Candida albicans* (Quaranta et al. 2011). All these organisms suffered extensive membrane damage by metallic Cu but their genetic materials were unaffected during the stress event prior to death. That Cu, both in its ionic and its metallic form, is not genotoxic is probably best documented by two observations. First, Cu ion stress did not cause mutations in *E. coli* (Macomber et al. 2007). Second, an organism with exceptional DNA-repair capabilities, such as *D. radiodurans*, was as efficiently inactivated by metallic Cu as *E. coli* (Espirito Santo et al. 2011) further disfavoring the DNA-damage hypothesis of Cu-mediated cell death. Furthermore, care has to be taken not to confuse the in vitro redox-activities of Cu with what is happening inside the cell. For example Cu had strong mutagenic properties when phage-DNA was in contact with Cu ions in vitro and the DNA was then transfected into *E. coli* (Tkeshelashvili et al. 1991). In contrast, when the toxic properties of Cu ions on living cells were studied in vivo recently, Cu damaged catalytic iron–sulfur clusters in essential proteins rather than DNA (Macomber and Imlay 2009).

Previous studies have demonstrated both the antimicrobial properties of ionic (e.g., Borkow and Gabbay 2004; De Muynck et al. 2010; Nie et al. 2010) and also metallic Cu surfaces against Staphylococci (Noyce et al. 2006; Airey and Verran 2007; Tolba et al. 2007) but did not offer a conclusive explanation for the mechanism of action of metallic Cu surfaces. Only recently an effort was made to elucidate the underlying reasons why Cu surfaces efficiently kill Staphylococci (Weaver et al. 2010). The authors claimed to have found two independent cellular targets of metallic Cu toxicity, DNA, and respiration. Conversely, little damaging effect on cell membrane integrity was observed. This is remarkable, because respiration is a process tied to the cytoplasmic membrane that depends on intact membranes for build-up and use of a proton-motive force across the membrane for ATP biosynthesis. It is hard to consolidate inhibition of respiration with little membrane damage. Certainly, it is possible but unlikely that the observed damage accrued only in the respiratory proteins embedded within the membrane but not in the membrane itself.

Along this line of argumentation it is noteworthy that *D. radiodurans* was killed on Cu surfaces as quickly as *E. coli* (Espirito Santo et al. 2011). *Deinococcus radiodurans* is resistant to oxidative protein carboxylation and can reconstitute genomes fragmented from exposure to ionizing radiation (Daly et al. 2007). Because *D. radiodurans* is nevertheless rapidly inactivated by metallic Cu, makes it unlikely that DNA-genotoxicity and lethal protein damage are the major mechanism-of-action of contact killing by Cu surfaces.

In one aspect, staphylococcal cells were clearly different from those of other bacteria tested previously on dry Cu. It took about seven times longer to kill *Staphylococcus* compared to *E. coli* or *D. radiodurans* (Espirito Santo et al. 2011). A prolonged killing-process was certainly due to the thick peptidoglycan of staphylococcal cell walls. This strong diffusion barrier might also account for the poor propidium iodide staining seen in Weaver et al. (2010) though in our hands we had little difficulties staining with this dye.

Our findings that *Staphylococcus* membranes were severely damaged upon contact with metallic Cu, propose the membrane as primary target of Cu surface-induced lethality. This notion is supported by our ICP-MS analysis. After 7 min of exposure to Cu, when the cells were completely killed, the intracellular Cu concentration had reached lower levels than at 5 min (Fig. 1B) indicative of membrane leakage. Also, because the membrane had become permeable to the dye propidium iodide, the membrane potential had dissipated and so too had respiration ceased. Previously, we had noticed that fluorescent dye staining gave nonreproducible results when performed directly on metallic Cu (Espirito Santo et al. 2011). Now we routinely remove cells from surfaces before staining. It might be that negative staining-artifacts accounted for the contradictory results reported in Weaver et al. (2010) and the patchy appearance of live and dead *S. aureus* cells in Airey and Verran (2007). However, it should be noted that these studies investigated moist Cu surfaces. Our study was concerned with dry Cu surfaces because such dry touch-surfaces may be encountered in public and clinical environments where Cu has recently been put to use (Casey et al. 2010; Mikolay et al. 2010). Nevertheless, contradictory results were presented for Enterococci on dry Cu surfaces recently (Warnes and Keevil 2011). While that study partially confirmed earlier work from our laboratory (Espirito Santo et al. 2008, 2011) the authors suggest DNA damage was among the first events of Cu surface mediated killing. In this competing model, membranes were not compromised at an initial early stage but only after cells were inactivated.

This study at hand suggests that killing of Staphylococci on dry metallic Cu surfaces follows the same principles as inactivation of other bacteria and yeasts. These results thus offer an alternative on the molecular mechanisms leading to cell death in these thick-cell-walled coccoid bacteria: genotoxicity may not be responsible for killing of the cells but rather a compromised cytoplasmic membrane leads to cessation of life processes.

Molecular knowledge of the mode-of-action exerted by metallic Cu on microbes is certainly not strictly necessary for widespread application of antimicrobial surfaces in hygiene-sensitive areas. Currently, it is agreed-upon that genomic material will eventually degrade on metallic Cu (Weaver et al. 2011; Warnes and Keevil 2010; Espirito Santo and Grass, unpublished observations) but it is controversial if this process is causative for or subsequent to cell death (Weaver et al. 2010; Espirito Santo et al. 2011). We propose that current data favor the model that membranes are damaged first, causing lethality, followed by protein oxidation (Nandakumar et al. 2011) and DNA-degradation. In depth understanding of the sensitive cellular targets of Cu toxicity and the order of events leading to death, however, can be expected to provide new opportunities for improving the efficacy of Cu surfaces against microbes.
